# Foundation model-enhanced multimodal radiomics for predicting response to chemo-immunotherapy in advanced lung squamous cell carcinoma

**DOI:** 10.3389/fonc.2026.1863080

**Published:** 2026-07-20

**Authors:** Zhichao Wang, Yang Zhang, Chuchu He, Meng Wang, Jun Cai

**Affiliations:** 1 Department of Oncology, The First Affiliated Hospital of Yangtze University, Jingzhou, China; 2 Hubei Provincial Clinical Research Center for Personalized Cancer Diagnosis and Therapy, Jingzhou, Hubei, China

**Keywords:** chemo-immunotherapy, foundation model, lung squamous cell carcinoma, radiomics, treatment response prediction

## Abstract

**Objective:**

To develop and validate a foundation model–driven multimodal fusion framework for non-invasive prediction of treatment response to first-line chemo-immunotherapy in patients with advanced lung squamous cell carcinoma (LUSC).

**Methods:**

In this retrospective study, baseline contrast-enhanced computer tomography (CT) images and clinical data from patients with advanced LUSC receiving first-line chemo-immunotherapy were collected. Handcrafted radiomics features were extracted from tumor regions of interest, and 2.5D deep learning features were extracted using a DINO-pretrained vision Transformer foundation model. Clinical variables were incorporated to construct a multi-source features fusion model based on machine learning classifiers. Model performance was evaluated using the area under the receiver operating characteristic curve (AUC), accuracy, sensitivity, and specificity. DeLong testing, decision curve analysis (DCA), net reclassification improvement (NRI), and integrated discrimination improvement (IDI) were performed for comparative assessment. Shapley additive explanations (SHAP) analysis was applied to enhance model interpretability and decision transparency.

**Results:**

Among the constructed models, the multi-source features fusion model (FusionModel) achieved the best predictive performance, with an AUC of 0.903 and an accuracy of 0.885 in the training cohort, and an AUC of 0.863 and an accuracy of 0.836 in the validation cohort. FusionModel outperformed models based on single-modality features and demonstrated superior net clinical benefit on DCA. NRI and IDI analyses further supported improved reclassification ability. SHAP analysis revealed that deep learning features contributed dominantly, while radiomics and clinical variables provided complementary prognostic information, supporting the biological plausibility of the model.

**Conclusion:**

The foundation model-driven multi-source features fusion model enabled accurate and interpretable prediction of chemo-immunotherapy response in advanced LUSC. This strategy demonstrated strong discriminative performance and clinical applicability, highlighting its potential as a non-invasive tool for individualized treatment stratification.

## 1 Introduction

Non-small cell lung cancer (NSCLC) represents the predominant histological subtype of lung cancer worldwide. Approximately 70%-80% of patients are diagnosed with stage III or IV NSCLC, with a five-year overall survival rate of only 15%-30% (1–3). In recent years, the advent of immune checkpoint inhibitors (ICIs) has substantially transformed the therapeutic landscape of advanced NSCLC, particularly for patients without actionable driver mutations. Compared with lung adenocarcinoma, advanced LUSC is characterized by a lower rate of identified driver gene mutations. Consequently, platinum-based chemotherapy combined with immune checkpoint inhibitors has become one of the main first-line treatment strategies for advanced LUSC (4). Despite these advances, clinical responses to chemo-immunotherapy remain heterogeneous. A considerable proportion of patients experience primary resistance, immune-related adverse events, or early disease progression, which complicates treatment decision-making and prognostic assessment (5). Currently available biomarkers, such as programmed death ligand-1 (PD-L1) expression, tumor mutational burden (TMB), and selected genomic alterations, provide limited predictive accuracy and fail to fully explain heterogeneity (4, 6, 7). Therefore, the identification of reliable and non-invasive biomarkers capable of stratifying patients according to therapeutic benefit is of critical importance for optimizing individualized treatment strategies.

Radiomics has emerged as a quantitative imaging approach that transforms medical images into high-dimensional data reflecting tumor intensity, shape, and texture patterns (8, 9). Several studies have demonstrated the potential of CT-based radiomics in predicting treatment response, survival outcomes, and toxicity in lung cancer (9–11). In particular, a review study highlighted the reliability and feasibility of CT-based radiomics in predicting immune-based therapy outcomes in patients with advanced NSCLC (12). However, traditional radiomics approaches rely on handcrafted feature engineering and predefined feature families, which may limit their ability to capture complex semantic patterns (8, 13). These limitations have impeded broader clinical translation and underscore the need for more robust and expressive feature extraction strategies.

To address these challenges, recent investigations have explored integrative modeling frameworks that combine deep learning features with radiomics and clinical variables (14). Deep learning models, particularly convolutional neural networks, can automatically learn hierarchical representations from medical images without reliance on manually designed features (15). Several studies have reported improved prediction performance when deep learning features are combined with radiomics and clinical information compared with single-modality models (16, 17).

Nevertheless, most deep learning models in this domain are either trained from scratch or pretrained on relatively limited medical datasets, which may introduce dataset-inherent bias and restrict generalizability (18, 19). Foundation models pretrained on large-scale datasets using self-supervised learning have recently demonstrated enhanced robustness and transferable semantic representations (20, 21). Leveraging such models may provide more stable and biologically meaningful imaging features. Furthermore, many prior outcome prediction studies have not adequately considered the biological heterogeneity between histological subtypes of NSCLC, including differences in genomic landscapes, tumor microenvironment characteristics, and therapeutic pathways. Predictive modeling specifically tailored to advanced LUSC remains relatively underexplored, despite its distinct biological and therapeutic profile.

Accordingly, the present study aimed to develop and validate a foundation model–driven radiomics framework for predicting the efficacy of first-line chemo-immunotherapy in patients with advanced LUSC. Foundation model features were integrated with radiomics features and clinical variables to construct a robust and interpretable multi-source prediction model. Finally, SHAP analysis was employed to enhance model interpretability by quantifying feature contributions at both global and individual levels.

## 2 Materials and methods

### 2.1 Patient cohorts

**Figure 1:**
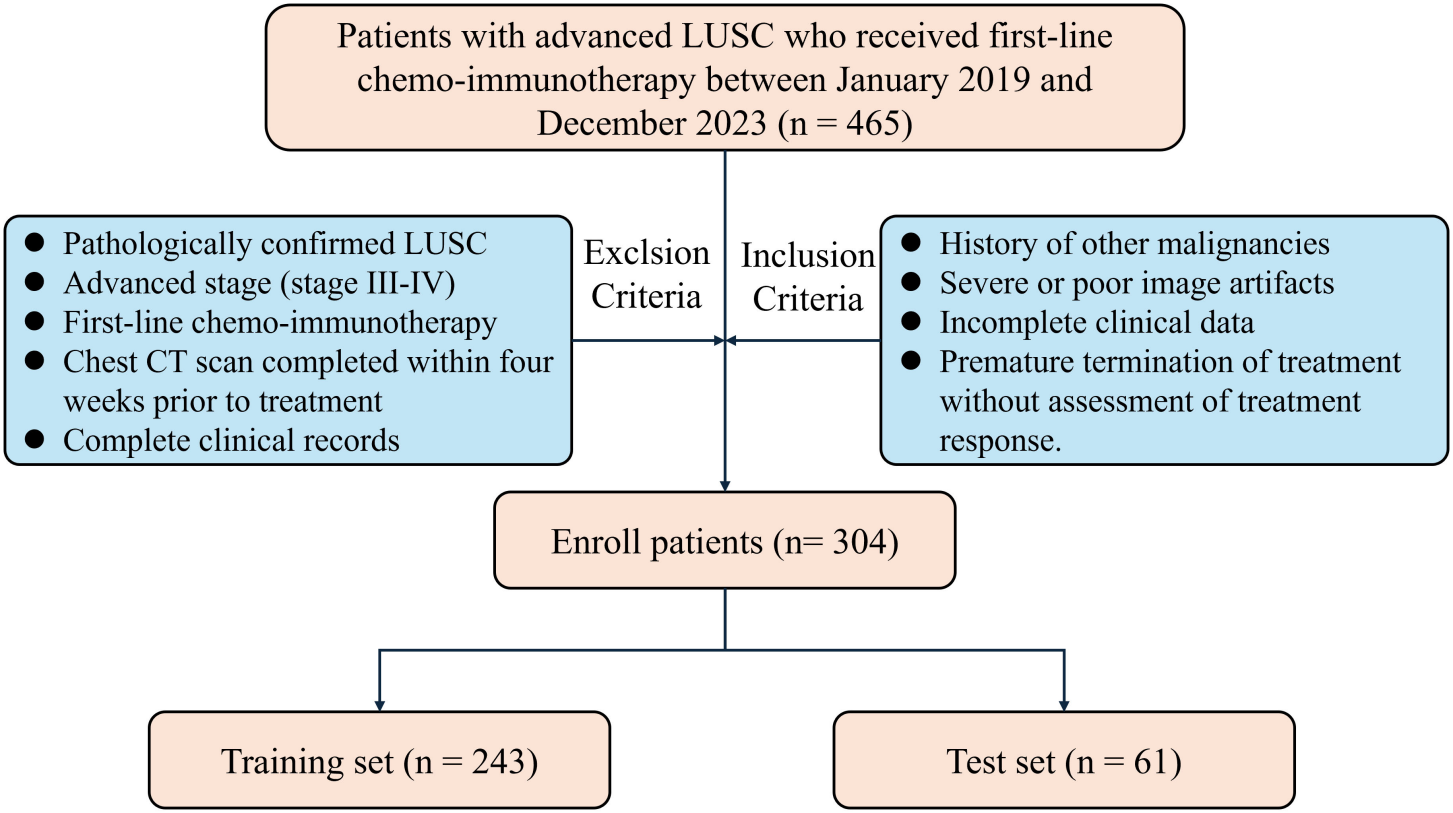
Patient inclusion and exclusion flowchart.

This retrospective study included 304 patients with advanced LUSC who received first-line chemo-immunotherapy at our hospital between January 2019 and December 2023. This study was approved by the institutional ethics committee, and informed consent was waived due to its retrospective nature. The patient enrollment flowchart is shown in **Figure 1**.

Inclusion criteria: (1) Pathologically confirmed squamous cell carcinoma of the lung; (2) Advanced stage (stage III-IV) according to the American Joint Committee on Cancer (AJCC) 8th edition staging system; (3) Received platinum-based chemotherapy combined with immune checkpoint inhibitors as first-line systemic therapy; (4) Had a chest CT scan completed within four weeks prior to treatment; (5) Complete clinical records and follow-up information. Exclusion criteria: (1) History of other malignancies; (2) Severe or poor image artifacts; (3) Incomplete clinical data; (4) Premature termination of treatment without assessment of treatment response.

A stratified sampling strategy was used to randomly assign patients to a training cohort (n = 243) and an independent validation cohort (n = 61) to maintain a similar proportion of effective and ineffective patients in both cohorts. Treatment response was assessed according to Response Evaluation Criteria in Solid Tumors (RECIST) v1.1 criteria. The primary endpoint was treatment response, evaluated according to RECIST 1.1 criteria. Patients were categorized into responders (complete response [CR], partial response [PR], and stable disease [SD], label = 1) and non-responders (progressive disease [PD], label = 0). This definition corresponds to the disease control rate (CR + PR + SD), a commonly used endpoint in solid tumor and immunotherapy-related studies, reflecting overall treatment benefit in terms of tumor burden stabilization or reduction (22–24).

### 2.2 CT acquisition and tumor segmentation

All patients underwent contrast-enhanced chest CT examinations prior to treatment. Portal venous phase CT Images were retrieved from the institution’s picture archiving and communication system (PACS) in digital imaging and communication in medicine (DICOM) format. Scanners used in this study included a Philips 128-slice Micro ICT and a GE 64-slice Gemstone CT, the scanning parameters are presented in **Supplementary Table 1**. For contrast-enhanced imaging, a non-ionic iodinated contrast agent (iohexol) was administered via a power injector at a dose of 1.5 ml/kg and an injection rate of 2.0 ml/s through an antecubital vein.

**Figure 2:**
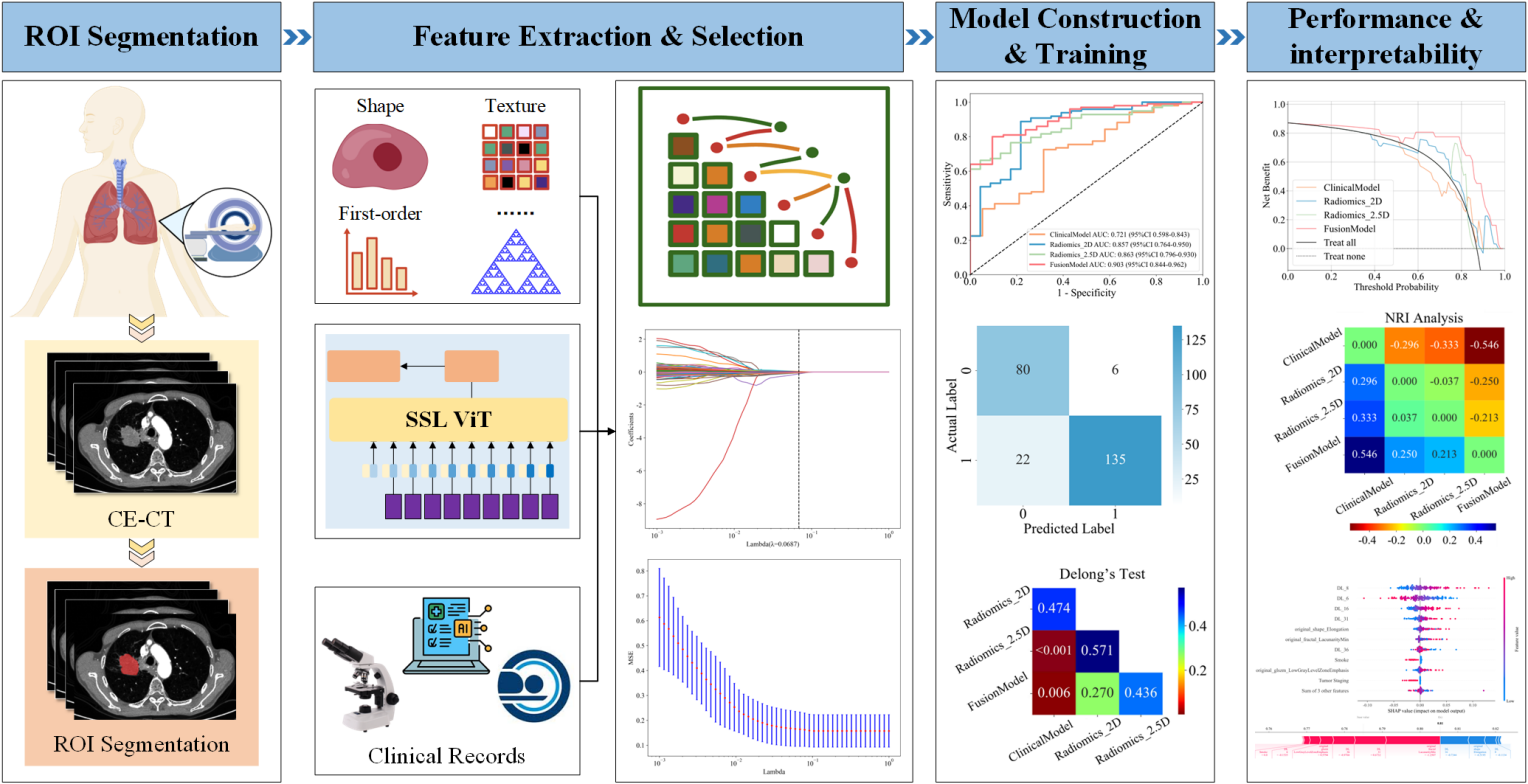
Overall study design.

CT images underwent a standardized preprocessing pipeline before feature extraction. Images were resampled to a voxel size of 1*1*5 mm³ using linear interpolation. After truncating intensity values within the range of -1400 to 200 Hounsfield Unit (HU), standard lung window settings were used (window width: 1500 HU, window level: -600 HU). Tumors were manually segmented using ITK-SNAP medical imaging software to generate three-dimensional regions of interest (ROI). Tumor delineation was independently performed by two physicians with over five years of experience in thoracic imaging, neither of whom were aware of the treatment response. All final ROIs were reviewed and confirmed by a senior thoracic radiologist with fifteen years of experience to ensure the segmentation accuracy and consistency. To evaluate segmentation reproducibility, an intraclass correlation coefficient (ICC) greater than 0.75 was applied to assess the inter-observer agreement of radiomics features (50 randomly selected cases). The overall workflow of this study is shown in **Figure 2**.

### 2.3 Treatment and outcome definition

All enrolled patients received first-line systemic chemotherapy-immunotherapy. Treatment regimens included platinum-based doublet chemotherapy combined with ICIs targeting the programmed death-1 (PD-1) or PD-L1 pathway. The chemotherapy backbone typically included platinum agents (cisplatin or carboplatin) combined with third-generation cytotoxic drugs such as paclitaxel, nab-paclitaxel, or gemcitabine. ICIs were administered intravenously at the standard dose and schedules specified in the respective prescribing information. Treatment typically consisted of 4–6 cycles, followed by maintenance immunotherapy when clinically indicated. Treatment adjustments were made at the discretion of the attending physicians based on treatment-related toxicities or disease progression to reflect real-world clinical practice.

Tumor response was assessed according to RECIST v1.1 criteria. Baseline CT scans were obtained before treatment initiation, followed by routine follow-up imaging every 6–8 weeks during therapy. All response evaluations were independently performed by two experienced physicians specializing in oncologic imaging assessment, who were blinded to clinical information and study outcomes. In cases of disagreement, a consensus was reached through joint review, and a senior radiologist with extensive experience in tumor response evaluation was consulted when necessary. The final treatment response was defined as the best overall response recorded during the entire treatment course.

### 2.4 Feature extraction and model construction

#### 2.4.1 Handcrafted radiomics features

Handcrafted radiomics features were extracted from the three-dimensional tumor ROIs on pretreatment CT images using a standardized radiomics pipeline. A total of 203 radiomics features were initially extracted for each patient, encompassing multiple feature categories, including shape descriptors, first-order statistical features, and texture features derived from the gray-level co-occurrence matrix (GLCM), gray-level run-length matrix (GLRLM), gray-level size-zone matrix (GLSZM), and gray-level dependence matrix (GLDM). In addition, higher-order features characterizing complex tumor patterns, such as Hessian matrix–based features, fractal dimension features, and topological descriptors, were included to comprehensively capture intratumoral heterogeneity. Detailed definitions and parameter settings of all radiomics features are provided in **Supplementary Table 2**.

#### 2.4.2 Foundation model–derived features

Pre-trained foundation models have been shown to learn robust image representations from large-scale datasets, which may help alleviate the limitations and potential biases associated with training deep learning models from scratch (25). In this study, the foundation model adopted was RadioDINO, a Vision Transformer (ViT) architecture trained using the DINO self-supervised learning framework, containing approximately 21 million parameters. Unlike conventional DINO models pretrained on natural image datasets, RadioDINO was pretrained on RadImageNet dataset, a large-scale medical imaging dataset comprising over one million radiological images, including CT data.

This medical imaging-specific pretraining may enable the model to learn generalizable radiology-oriented representations and capture high-level imaging patterns such as anatomical structures, texture heterogeneity, and spatial intensity variations (26). These transferable representations support effective feature extraction across downstream medical imaging tasks, even without task-specific fine-tuning. In this study, RadioDINO was used as a frozen feature extractor, and no fine-tuning or parameter updates were performed on the foundation model. This strategy preserves the pretrained radiological knowledge and reduces the risk of overfitting in relatively small datasets.

Deep learning features were extracted from segmented ROIs. To ensure reproducibility, the segmented ROIs were adjusted to a fixed 256*256 pixels. The 2.5D input consisted of the axial slice with the largest tumor cross-sectional area and its two adjacent slices above and below it. Therefore, the feature vectors of the five slices were concatenated to obtain a 1920-dimensional deep learning feature representation (384 * 5) for each patient. To reduce feature redundancy and alleviate overfitting, we applied principal component analysis (PCA) to the concatenated feature vectors and retained the first 64 principal components as the final deep learning feature representation.

#### 2.4.3 Feature selection and model construction

Six commonly available clinical variables were included, including gender, age, smoking history, tumor stage, distant metastasis, and chronic lung disease, based on their potential association with tumor biology, disease burden, and host immune status. All extracted features were standardized using Z-score normalization to eliminate scale differences. A two-step feature selection strategy was employed to reduce dimensionality and enhance model robustness. First, Pearson correlation analysis was performed to identify and remove highly correlated features, with a threshold of 0.9. Subsequently, the Least Absolute Shrinkage and Selection Operator (LASSO) method with 10-fold cross-validation was used to further filter features while preventing overfitting. Multiple predictive models were constructed using different combinations, including models based on traditional radiomics, models based on deep learning features, and fusion models. The selected machine learning models included Light Gradient Boosting Machine (LightGBM), Random Forest (RF), k-Nearest Neighbor (KNN), Extreme Gradient Boosting (XGBoost), and Support Vector Machine (SVM). All models were optimized for hyperparameters on the training cohort and then evaluated on the validation cohort to assess their prediction performance.

### 2.5 Model evaluation

This study used multiple metrics to comprehensively evaluate model performance, including AUC, accuracy, sensitivity (SEN), specificity (SPE), and the DeLong test for ROC curve comparison. We performed DCA to assess the clinical usability of the models and used NRI and IDI to assess performance improvements and changes in discrimination. We used p-values to assess the statistical significance of IDI. Furthermore, SHAP analysis was employed to quantify feature contributions at both the global and individual levels, thereby enhancing model interpretability.

### 2.6 Statistical analysis

Statistical analyzes were performed using Python (version 3.7.12) and SPSS software (version 26.0). Continuous variables were expressed as mean ± standard deviation, and categorical variables were expressed as counts and percentages. Continuous variables were analyzed using Student’s t-test or Mann-Whitney U test, while categorical variables were compared using chi-square test or Fisher’s exact test, depending on the specific circumstances. The model performance was primarily evaluated using the AUC. The DeLong test was used to compare the AUC values between different models. DCA was used to assess the net clinical benefit of each model at different threshold probabilities. All statistical tests were two-tailed, and a p-value less than 0.05 was considered statistically significant.

## 3 Results

### 3.1 Patient characteristics

This retrospective study included 304 patients, who were randomly assigned in an approximately 8:2 ratio to the training cohort (n = 243) and the validation cohort (n = 61). Baseline demographic characteristics of the patients are shown in **Table 1**. The mean age of this cohort was 60.78 ± 8.02 years, predominantly male (84.21%), with 48 female patients (15.79%). Most patients had a history of smoking (85.53%). In the training cohort, 86 patients (35.4%) were non-responders (labeled 0), and 157 patients (64.6%) were responders (labeled 1). In the validation cohort, 22 patients (36.1%) were non-responders, and 39 patients (63.9%) were respondents. There were no statistically significant differences between the training and validation cohorts in terms of age, sex, clinical stage, smoking status, tumor location, treatment outcome, or chronic lung disease (all P > 0.05).

**Table 1:** Patient characteristics.

Characteristics	Total (n = 304)	Training cohort (n = 243)	Validation cohort (n = 61)	*P* value
Age	60.78 ± 8.02	60.91 ± 7.46	60.28 ± 8.62	0.243
Gender				0.521
Male	256 (84.21%)	202 (83.13%)	54 (88.52%)	
Female	48 (15.79%)	41 (16.87%)	7 (11.48%)	
Clinical stage				0.292
III	149 (49.01%)	118 (48.56%)	31 (50.82%)	
IV	155 (50.99%)	125 (51.44%)	30 (49.18%)	
Smoking				0.575
Current/Former	260 (85.53%)	209 (86.01%)	51 (83.61%)	
Never	44 (14.47%)	34 (13.99%)	10 (16.39%)	
Tumor location				0.194
Left	146 (48.03%)	118 (48.56%)	28 (45.90%)	
Right	158 (51.97%)	125 (51.44%)	33 (54.10%)	
Outcome				0.428
CR/PR/SD	196 (64.47%)	157 (64.61%)	39 (63.93%)	
PD	108 (35.53%)	86 (35.39%)	22 (36.07%)	
Chronic lung disease				0.117
Positive	157 (51.64%)	120 (49.38%)	37 (60.66%)	
Negative	147 (48.36%)	123 (50.62%)	24 (39.34%)	

### 3.2 Performance of conventional radiomics models with different machine learning classifiers

**Figure 3:**
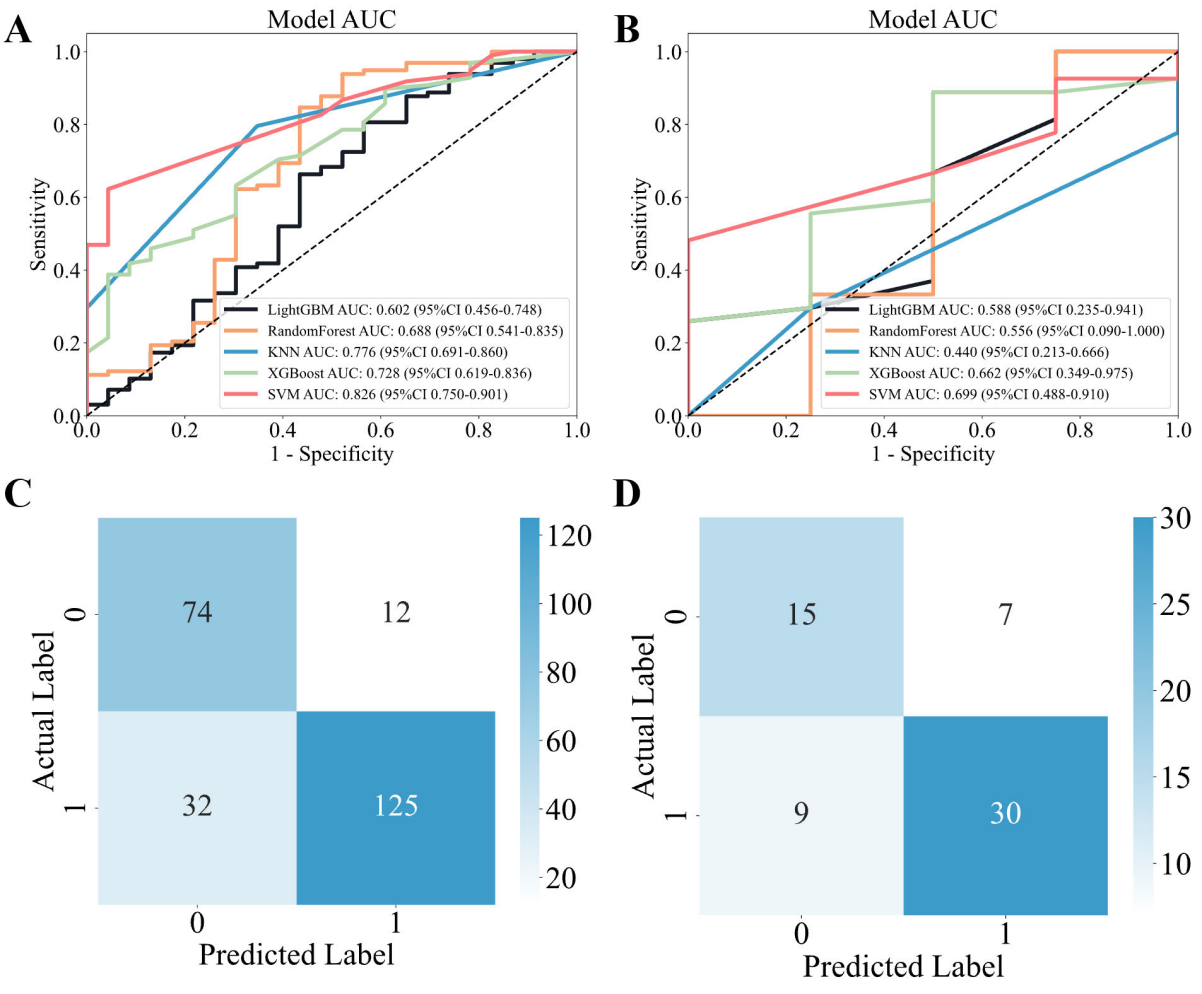
Results of traditional radiomics models. ROC curves of five machine learning models on the training **(A)** and validation cohorts **(B)**. Confusion matrix of SVM model on training **(C)** and validation cohorts **(D)**.

In this study, five commonly used machine learning classifiers were trained and evaluated based on 203 handcrafted radiomics features extracted from pre-treatment CT images. All models were trained and hyperparameters optimized on a training cohort, followed by evaluation on validation cohort, as shown in **Table 2** and **Figure 3**.

**Table 2:** Performance comparison of different machine learning models.

Model	Cohorts	AUC	ACC	SEN	SPE
LightGBM	Training	0.602 (0.456-0.748)	0.733	0.803	0.605
	Validation	0.588 (0.235-0.941)	0.639	0.538	0.818
RandomForest	Training	0.688 (0.541-0.835)	0.687	0.618	0.814
	Validation	0.556 (0.090-1.000)	0.557	0.513	0.636
KNN	Training	0.776 (0.691-0.860)	0.770	0.803	0.709
	Validation	0.440 (0.213-0.666)	0.557	0.462	0.727
XGBoost	Training	0.728 (0.619-0.836)	0.778	0.758	0.814
	Validation	0.662 (0.349-0.975)	0.639	0.692	0.545
SVM	Training	0.826 (0.750-0.901)	0.819	0.796	0.860
	Validation	0.699 (0.488-0.910)	0.738	0.769	0.682

Among the five classifiers, the SVM-based radiomics model achieved the best overall performance (**Figures 3A, B**). In the training cohort, the SVM model demonstrated superior predictive ability compared to the other four classifiers, with an AUC of 0.826 (95% CI: 0.750-0.901). More importantly, in the validation cohort, the SVM model achieved an AUC of 0.699 (95% CI: 0.488-0.910), exhibiting the best generalization ability and robustness. The confusion matrices are shown in **Figures 3C, D**. While other models achieved relatively high specificity or sensitivity, SVM demonstrated the most balanced performance across all metrics.

To further statistically evaluate differences between classifiers, pairwise comparisons of AUC were performed using DeLong’s test, and the results are presented in **Supplementary Figure 1**. The analysis showed that the SVM model achieved statistically significant superiority over KNN and LightGBM in the validation cohort, while differences compared with RandomForest and XGBoost did not reach statistical significance, indicating that SVM exhibited an overall but not absolute advantage across all comparisons. Therefore, based on its balanced performance and overall generalization ability across both cohorts, the SVM classifier was selected as the subsequent multi-source feature fusion model.

### 3.3 Comparison of clinical, radiomics, and deep learning-enhanced models

In this study, four models were further constructed to evaluate the incremental value of foundation model-derived deep learning features and clinical variables: (1) ClinicalModel, with 6 clinical variables, (2) Radiomics_2D, integrating radiomics features with 2D deep learning features; (3) Radiomics_2.5D, combining radiomics features with 2.5D deep learning features, and (4) FusionModel, integrating radiomics features, 2.5D deep learning features, and clinical variables.

**Figure 4:**
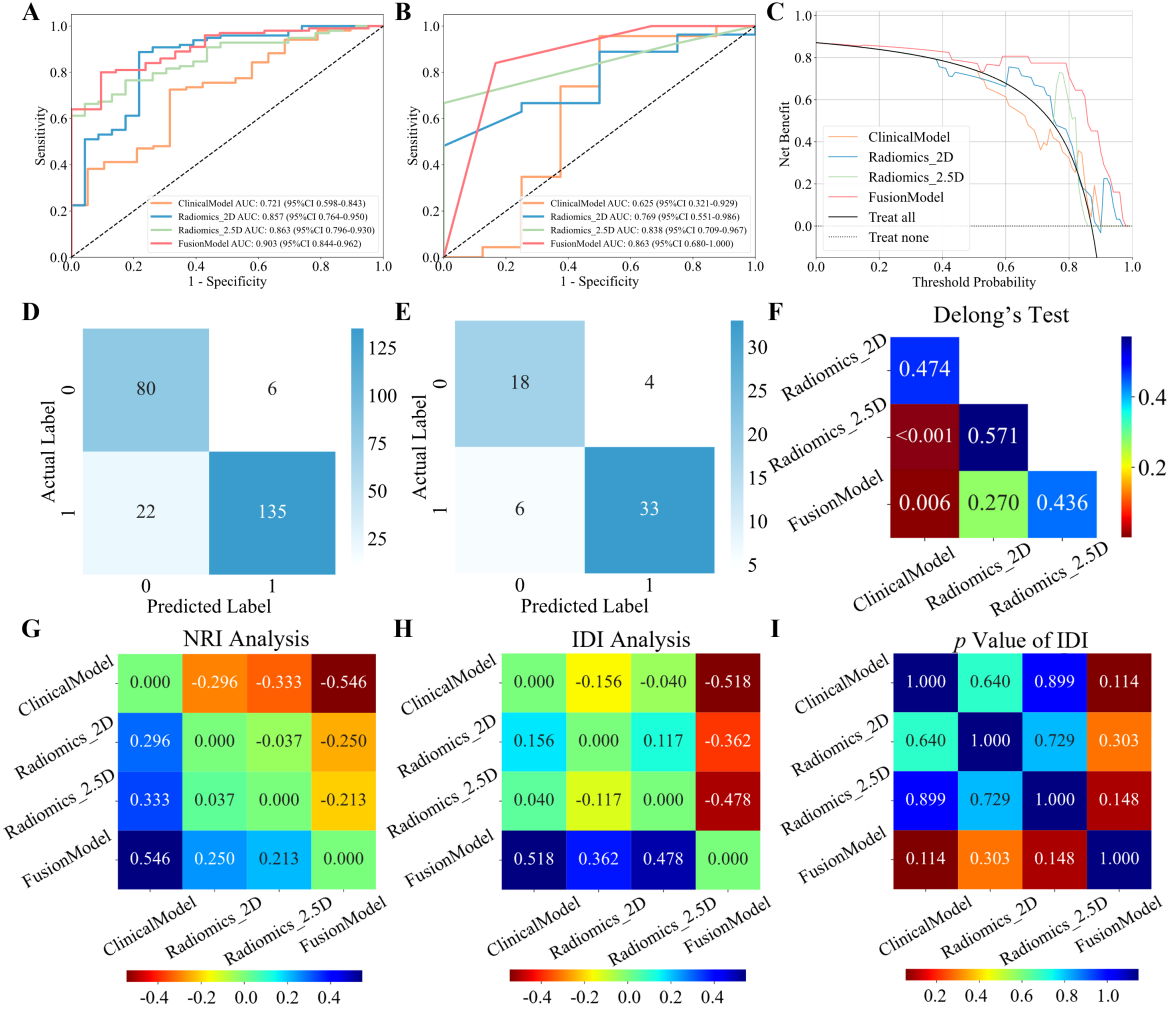
Evaluation of model prediction results. The ROC curves of the models on the training **(A)** and validation **(B)** cohorts. Decision curve analysis (DCA) showed that FusionModel provided higher net clinical benefit **(C)** across a wide range of threshold probabilities. The confusion matrix demonstrated the balanced classification of FusionModel on the training **(D)** and validation **(E)** cohorts. The DeLong test **(F)** indicated that FusionModel and Radiomics_2.5D significantly outperformed ClinicalModel. NRI **(G)** and IDI **(H)** analyses consistently showed that FusionModel outperformed the other models in reclassification, and both NRI and IDI values were positive, although the p-value of IDI **(I)** had limited statistical significance in pairwise comparisons.

The detailed results are presented in **Table 3** and **Figure 4**. The ClinicalModel demonstrated limited predictive ability, indicating that conventional clinical variables were insufficient for prediction of treatment response. The integration of deep learning features with radiomics features significantly improved predictive performance. Notably, Radiomics_2.5D outperformed Radiomics_2D in both AUC and accuracy, suggesting that incorporating contextual information from ROI provided complementary tumor characteristics.

**Table 3:** Comparison of prediction performance across different models.

Model	Cohorts	AUC	ACC	SEN	SPE
ClinicalModel	Training	0.721 (0.598-0.843)	0.712	0.732	0.674
	Validation	0.625 (0.321-0.929)	0.672	0.615	0.773
Radiomics_2D	Training	0.857 (0.764-0.950)	0.835	0.796	0.907
	Validation	0.769 (0.551-0.986)	0.738	0.795	0.636
Radiomics_2.5D	Training	0.863 (0.796-0.930)	0.860	0.847	0.884
	Validation	0.838 (0.709-0.967)	0.803	0.744	0.909
FusionModel	Training	0.903 (0.844-0.962)	0.885	0.860	0.930
	Validation	0.863 (0.680-1.000)	0.836	0.846	0.818

More importantly, the FusionModel achieved the best overall performance in both the training and validation cohorts, with AUCs of 0.903 (95% CI: 0.844-0.962) and 0.863 (95% CI: 0.680-1.000) respectively. Overall, the results in **Figure 4** collectively indicate that combining 2.5D deep learning and clinical features with radiomics features substantially enhanced predictive performance and clinical applicability.

### 3.4 SHAP-based model interpretability analysis

**Figure 5:**
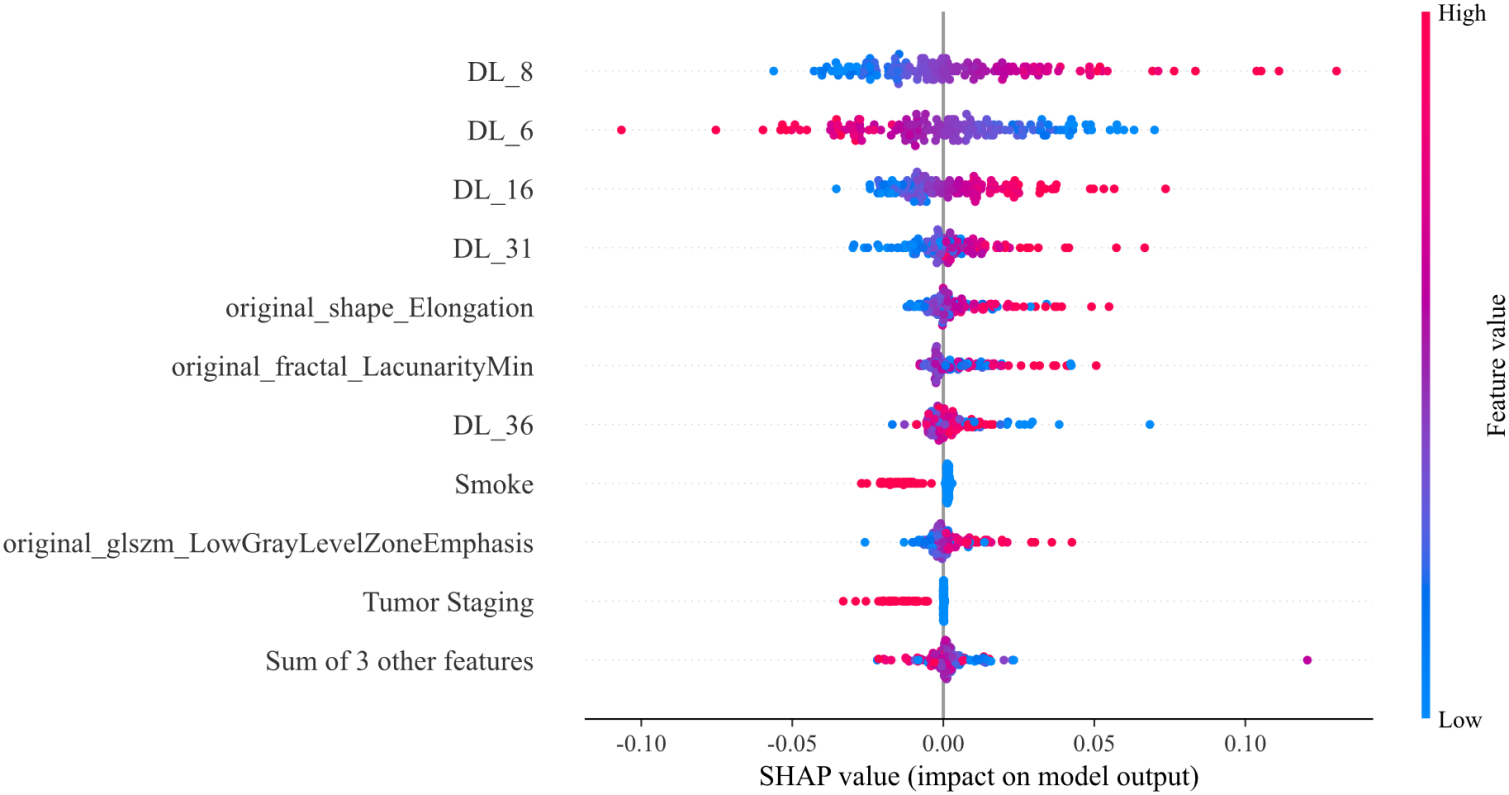
The SHAP summary plot of FusionModel provide an intuitive visualization of feature contributions. This figure reveals the contributions of multi-source imaging features to the prediction of treatment efficacy, utilizing color coding to distinguish between positive and negative predictive outcomes.

To improve model interpretability and explore the contribution of features to treatment response prediction, this study performed a SHAP analysis on FusionModel. As shown in **Figure 5**, the SHAP summary plot reveals that deep learning features contributed the most to the predictions, with DL_8, DL_6, and DL_16 being the top three most influential variables, indicating that the feature representations extracted by the foundation model captured information-rich tumor features relevant to treatment response. Several handcrafted radiomics features, including original_shape_Elongation, original_fractal_LacunarityMin, and original_glszm_LowGrayLevelZoneEmphasis, also showed significant contributions. For example, increased tumor elongation and specific texture heterogeneity patterns had measurable effects on prediction, reflecting the role of tumor morphology and intratumoral heterogeneity in modulating the efficacy of chemotherapy-immunotherapy regimens. Furthermore, the contributions of clinical variables such as smoking status and tumor stage were not negligible, suggesting that while clinical factors alone are insufficient for accurate differentiation, they can provide complementary prognostic information when combined with radiological features.

**Figure 6:**
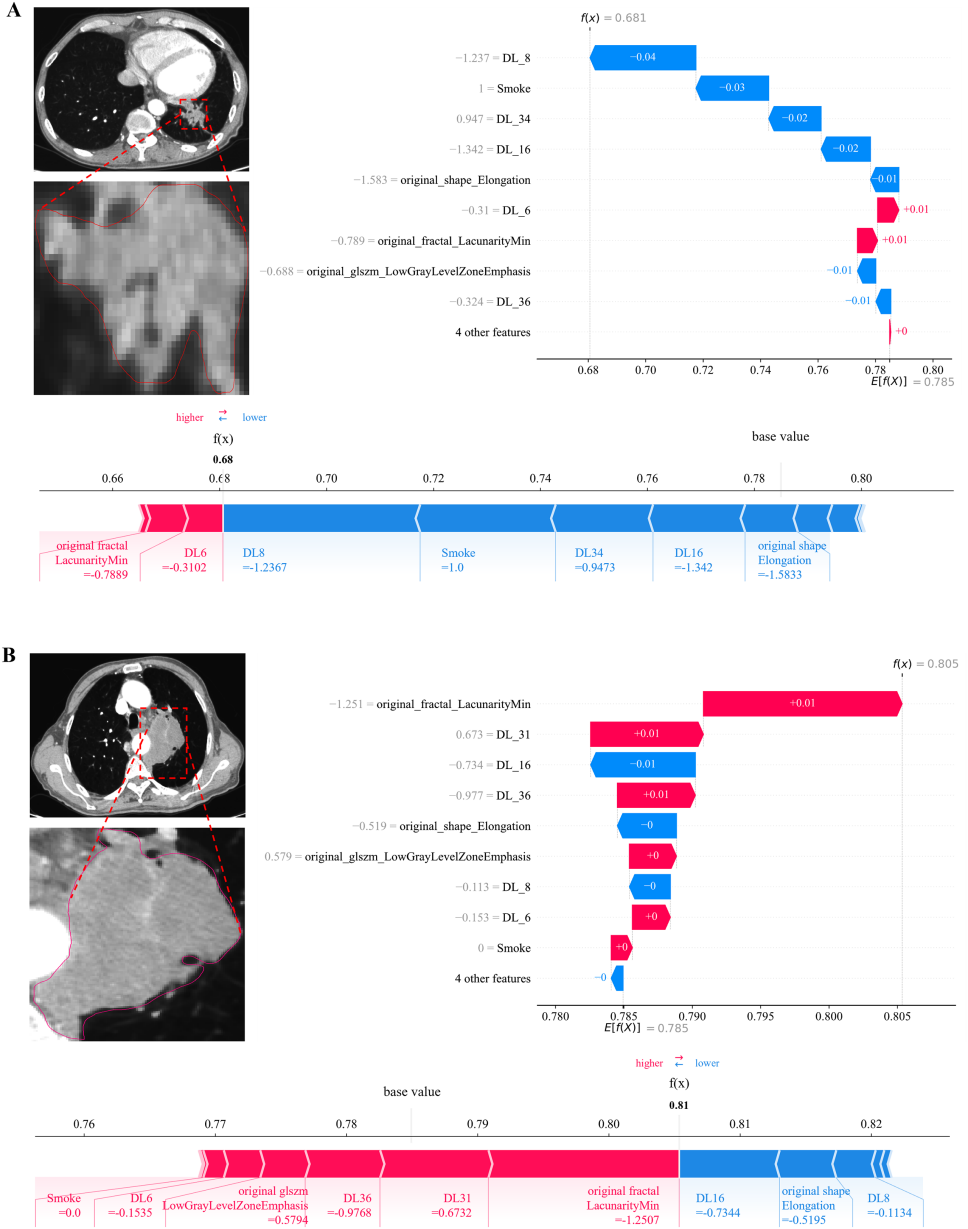
ROI visualizations, waterfall plots, and force plots for patients with labels 0 **(A)** and 1 **(B)**. Two representative samples demonstrate the role of key multi-source fusion features in predicting the efficacy of first-line chemo-immunotherapy for advanced LUSC.

Finally, **Figure 6** provides two representative SHAP force plot results. This demonstrates that the FusionModel achieves predictions through a dynamic balance of multimodal features, rather than relying on a single dominant factor, further supporting its biological rationale and interpretability at the patient level.

Although SHAP analysis can quantify the relative importance of features, it cannot directly explain the biological or radiological meaning of deep learning features. These latent representations are learned automatically by the foundation model through self-supervised pretraining rather than being explicitly designed to describe specific imaging characteristics. Consequently, SHAP indicates how much each latent feature contributes to the prediction but does not identify the specific imaging phenotype. Overall, the SHAP analysis confirms that the superior performance of the FusionModel stems from the integration of deep learning features, radiomics features, and clinical variables. This multimodal integration enables the model to capture high-level semantic representations and interpretable quantitative imaging features, thereby improving predictive performance and biological rationale.

## 4 Discussion

In this study, a foundation model-enhanced multimodal radiomics framework was developed and validated for predicting response to first-line chemo-immunotherapy in advanced LUSC. The proposed model achieved superior predictive performance compared with single-modal models (AUC = 0.863, accuracy =0.836). These results suggest that integrating radiomics, deep learning-derived features, and clinical variables can improve non-invasive prediction and pre-treatment stratification. Methodologically, foundation model-derived representations provide complementary information beyond handcrafted radiomics and clinical features, enhancing overall model robustness and stability.

Radiomics has been widely explored as a non-invasive approach for quantifying tumor heterogeneity and has shown potential in predicting treatment response and survival outcomes (27–29). However, most traditional radiomics methods rely on manually engineered features that primarily capture predefined characteristics, which may not fully reflect the high-level semantic and spatial complexity of tumor biology (30). In addition, radiomics features are sensitive to variations in image acquisition, segmentation, and reconstruction protocols, which may limit their generalizability. More importantly, handcrafted radiomics lacks the ability to learn task-specific hierarchical representations, thereby restricting its capacity to capture complex imaging phenotypes associated with chemo-immunotherapy response (8, 31).

To address these limitations, recent studies have proposed integrating radiomics with deep learning-based representations or multi-modal clinical information to improve predictive performance (32). In this context, multimodal fusion frameworks and foundation model-derived imaging features may provide complementary information by capturing both low-level quantitative descriptors and high-level semantic representations. Previous studies based on CT radiomics for predicting immunotherapy response in NSCLC have generally reported AUC values ranging from approximately 0.70 to 0.83, while more advanced deep learning or multimodal approaches have achieved AUC values around 0.80 to 0.87 in internal or external validation cohorts (12). Notably, the performance of our model (AUC = 0.863) lied within the upper range of previously reported results, suggesting competitive performance in LUSC-specific cohorts and indirectly supporting the reliability of the proposed feature representations.

In this study, a multimodal fusion framework was developed to capture complementary information from different data sources for predicting response to chemo-immunotherapy in advanced LUSC. Each modality provided distinct but complementary perspectives, radiomics quantified predefined tumor intensity, texture, and morphological characteristics, 2.5D deep learning features captured spatially contextual and higher-level imaging representations, and clinical variables provided patient-level prognostic context.

Importantly, the incorporation of a foundation model further enhanced the representational capacity of the deep learning branch. Unlike traditional supervised deep learning models trained from scratch, the foundation model pretrained on large-scale medical imaging data via self-supervised learning is capable of learning more transferable and semantically enriched imaging representations, mitigating potential dataset-specific bias (33, 34). This pretraining strategy based on large-scale datasets may improve feature robustness, as suggested by consistent performance improvements across single-modality and multimodal comparisons within our cohort, and alignment with previously reported performance ranges. The observed superior performance of the fusion model suggested that these heterogeneous features exhibited a synergistic effect rather than simple concatenation, enabling more comprehensive characterization of tumor phenotype and treatment response patterns. This multimodal integration therefore improved predictive performance and clinical applicability, highlighting the incremental value of foundation model-derived representations within a radiomics-based predictive framework.

Unlike lung adenocarcinoma, advanced LUSC lacks well-established targeted therapies and predominantly relies on chemo-immunotherapy as first-line treatment. In addition, LUSC exhibits a higher tumor mutational burden and distinct immune infiltration patterns, which may lead to heterogeneous response mechanisms to immunotherapy (35, 36). Therefore, this study focused on advanced LUSC to ensure biological and treatment homogeneity within the research cohort. This histology-specific design not only reduces potential confounding introduced by mixed subtypes but also addresses the relative paucity of predictive models specifically to LUSC, thereby enhancing model consistency, interpretability, and clinical relevance.

To enhance decision transparency, SHAP analysis was performed to reveal the underlying mechanisms contributing. The SHAP summary plot demonstrated that deep learning features (e.g., DL_8, DL_6, and DL_16) played dominant roles, indicating that latent representations extracted by the foundation model served as key drivers of predictive performance. Although these latent features cannot be directly mapped to explicit biological meanings, they can be learned automatically through self-supervised pretraining on large-scale medical imaging data. Therefore, they are expected to encode complex imaging patterns related to tumor morphology, intratumoral heterogeneity, boundary complexity, density distribution, or contextual spatial relationships that may not be fully captured by handcrafted radiomics features. Morphological radiomics features such as *original_shape_Elongation* reflected tumor geometric irregularity, which may be associated with invasive growth patterns and treatment sensitivity. Fractal-based features (*original_fractal_LacunarityMin*) characterized structural heterogeneity and spatial gap distribution, indirectly capturing tumor microenvironmental complexity. In addition, *original_glszm_LowGrayLevelZone Emphasis* related to intratumoral density distribution and potential necrotic components. Regarding clinical variables, smoking status had been associated with increased tumor mutational burden and neoantigen load, while tumor stage reflected overall disease burden and had been linked to alterations in the tumor immune microenvironment (37–39). Furthermore, SHAP force plots demonstrated that predictions were not driven by a single variable but rather resulted from a dynamic balance among multi-source features.

This interpretable integration of heterogeneous features enhanced clinician confidence and supported the translational potential of the model for individualized response prediction in patients with advanced LUSC receiving chemo-immunotherapy. Although SHAP analysis can quantify the relative contribution of foundation model-derived features to model predictions, these latent representations do not directly correspond to predefined radiological descriptors. Therefore, their interpretation should be viewed as contribution-based rather than strictly semantic. Improving the explainability of foundation model representations remains an important challenge and an active area of research in medical imaging artificial intelligence.

It should be noted that the foundation model used in this study was pretrained on a large-scale medical imaging dataset rather than natural image datasets. Therefore, the extracted deep learning features are expected to encode radiological semantic information that may be more relevant to tumor characterization and treatment response assessment. Nevertheless, systematic comparisons across different medical foundation models and adaptation strategies may provide further insights into the optimal use of foundation models for precision oncology and warrant future investigation.

This study also has several limitations. First, although there were no clinical missing values in this study, it was a retrospective single-center study with a relatively small sample size. In the future, it will benefit from multi-center data and efficient missing value handling methods. Second, the model is based solely on baseline pre-treatment CT images and does not include longitudinal imaging data reflecting dynamic changes in tumors during chemotherapy-immunotherapy. Third, the current model framework does not include other potential sources of information, such as histopathological features, genomic alterations, or transcriptomic profiles. Therefore, future research should focus on prospective, multi-center validation, incorporating longitudinal imaging data, and integrating multimodal molecular information to enhance the clinical applicability and biological interpretability of the predictive model.

## 5 Conclusion

In summary, this study developed and validated a multi-source feature fusion framework based on a foundation model for non-invasive prediction of chemotherapy-immunotherapy response in LUSC patients. By integrating radiomics features, 2.5D deep learning features, and clinical variables, the predictive model achieved optimal discriminative performance and demonstrated superior clinical applicability compared to single-modal methods. SHAP-based interpretability analysis further elucidated the complementary roles of deep learning, radiomics, and clinical features, enhancing the model’s transparency and biological rationale. In conclusion, these findings suggest that multi-modal feature integration based on a fundamental model holds promise as a robust and clinically valuable tool for personalized treatment stratification in advanced squamous cell carcinoma of the lung.

## Data Availability

The raw data supporting the conclusions of this article will be made available by the authors, without undue reservation.
