# Supplementary material for: Foundation model-enhanced multimodal radiomics for predicting response to chemo-immunotherapy in advanced lung squamous cell carcinoma
Source: Front Oncol. 2026 Jul 20;16:1863080. doi: 10.3389/fonc.2026.1863080 (PMC13429756; doi:10.3389/fonc.2026.1863080)
Supplement: Supplementary file 1 [file DataSheet1.docx]

Table S1 CT Scanners Parameters

| **Parameters** | **Philips 128-slice Micro ICT** | **GE 64-slice Gemstone CT** |
| --- | --- | --- |
| Acquisition type | Helical | Helical |
| Tube current | 100 - 200 mA (automatic exposure control) | |
| Tube voltage | 120 kVp | 120 kVp |
| Slice thickness | 5 mm | 5 mm |
| Image matrix | 512*512 | 512*512 |
| Pitch | 0.75-1.2 | 1.2-1.5 |
| Collimation | 0.6 mm | 0.6 mm |

Table S2 Radiomics features extracted in this study

| **Feature Type** | **Number** |
| --- | --- |
| **Hessian Matrix** | 39 |
| **Fractal Dimension** | 26 |
| **Topology** | 31 |
| **Shape** | 14 |
| **First-Order statistics** | 18 |
| **Texture** |  |
| -Gray Level Co-occurrence Matrix (GLCM) | 24 |
| -Gray Level Dependence Matrix (GLDM) | 14 |
| -Gray Level Run-Length Matrix (GLRLM) | 16 |
| -Gray Level Size Zone Matrix (GLSZM) | 16 |
| -Neighborhood Gray Tone Difference Matrix (NGTDM) | 5 |


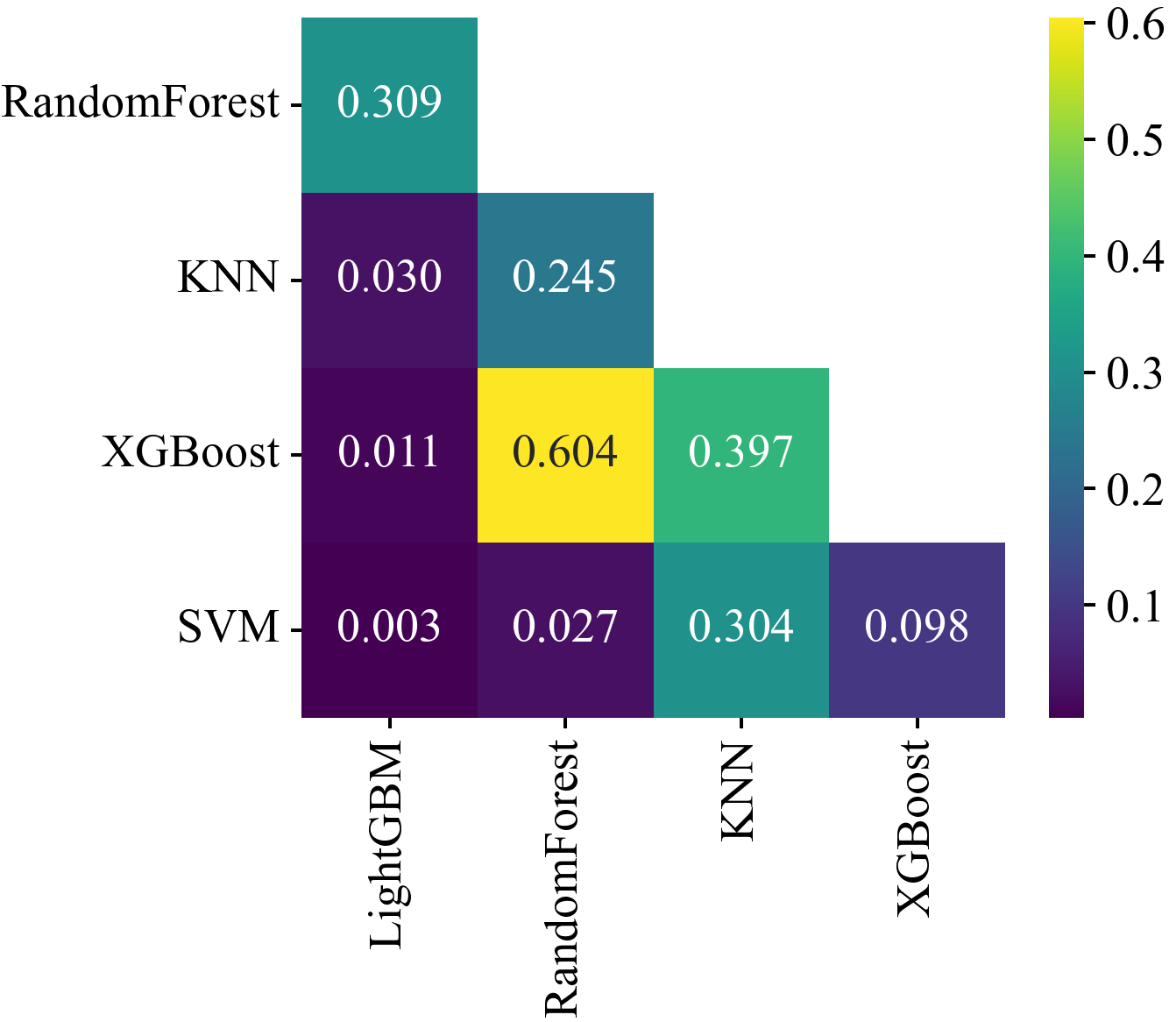

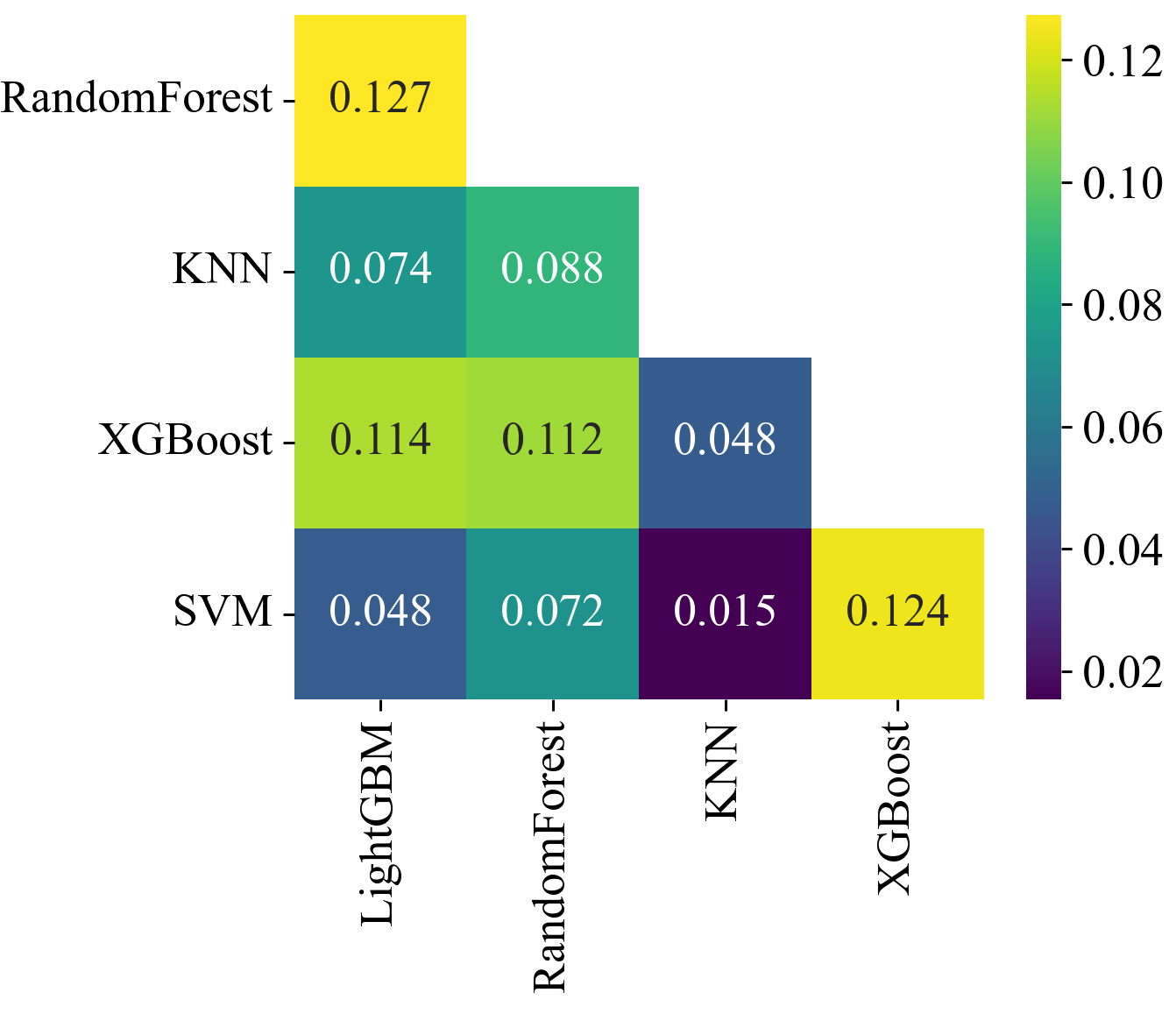


(1) Training cohort (2) Validation cohort

Figure S1 DeLong’s test results comparing AUC differences among five machine learning classifiers
